# Editorial: Enhancing the biological activity and applications of polysaccharides through modification

**DOI:** 10.3389/fnut.2026.1905245

**Published:** 2026-06-26

**Authors:** Kit-Leong Cheong, Eric Biney, Baoming Tian, Zichao Wang

**Affiliations:** 1 Guangdong Provincial Key Laboratory of Aquatic Product Processing and Safety, College of Food Science and Technology, Guangdong Ocean University, Zhanjiang, China; 2 College of Food Science and Technology, Zhejiang University of Technology, Huzhou, China; 3 School of Biological Engineering, Henan University of Technology, Zhengzhou, China

**Keywords:** application, bioactivity, modification, polysaccharides, structure

Polysaccharides are structurally diverse biopolymers with important nutritional, pharmacological, and technological potential. Their biological activities, including antioxidant, anti-inflammatory, immunomodulatory, hypoglycemic, anti-aging, and gut microbiota-regulating effects, are closely associated with molecular weight, monosaccharide composition, glycosidic linkage pattern, branching degree, charge density, functional groups, and conformational features. However, many native polysaccharides show limited solubility, low bioavailability, structural heterogeneity, and modest biological activity, which restrict their broader application in functional foods, nutraceuticals, and medicine. Therefore, modification strategies have become essential tools for improving polysaccharide functionality and for clarifying structure–activity relationships.

This Research Topic, “*Enhancing the biological activity and applications of polysaccharides through modification*,” brings together studies that demonstrate how extraction-assisted remodeling, microbial fermentation, chemical derivatization, selenylation, enzymatic-ultrasonic processing, and oligosaccharide-based intervention can reshape polysaccharide structures and enhance their biological performance. Rather than presenting modification as a simple processing step, the collected articles highlight modification as a rational approach to tune molecular architecture, improve bioactivity, and expand application scenarios.

**Figure 1:**
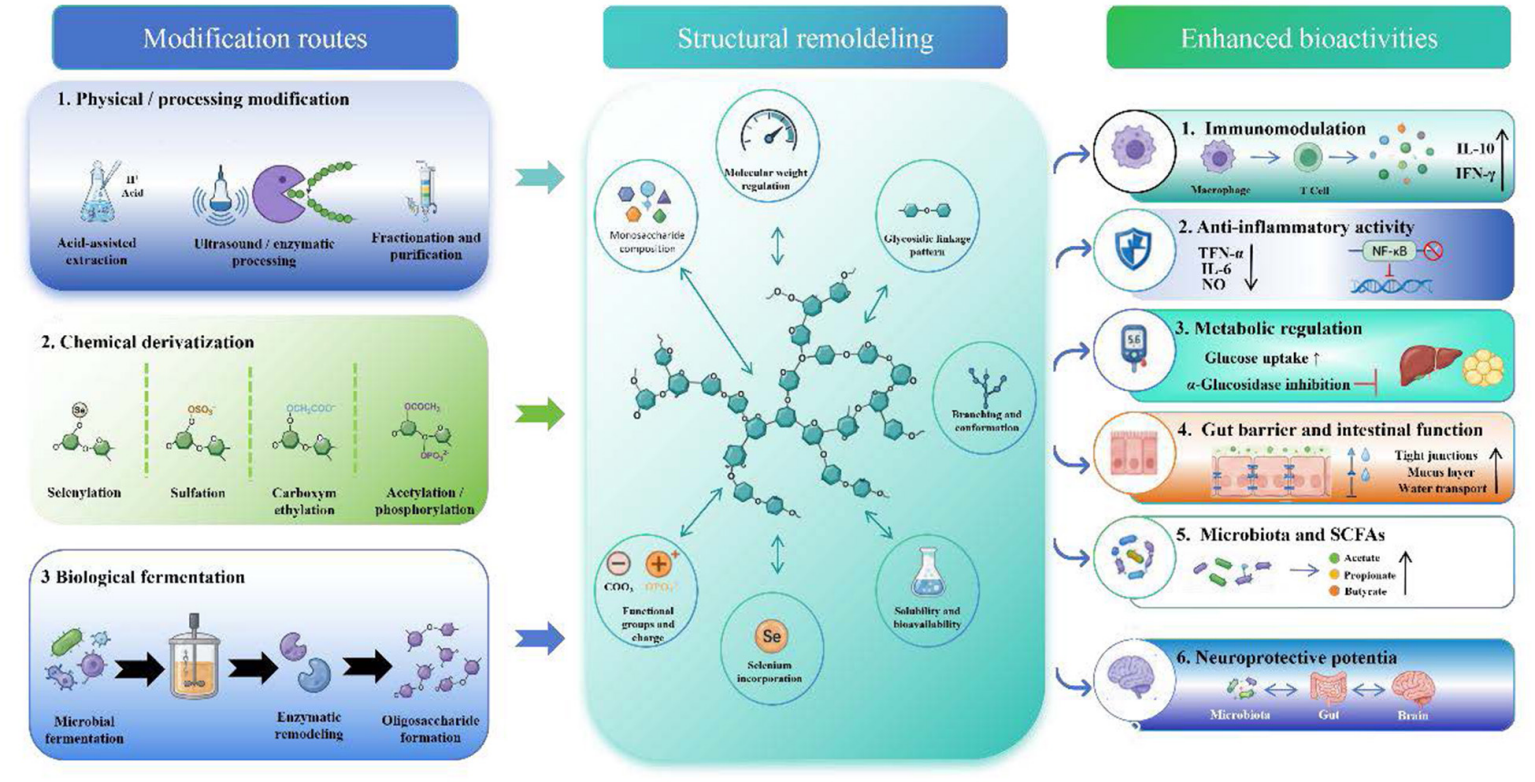
Modification-driven enhancement of polysaccharide bioactivity.

As summarized in Figure 1, the enhancement of polysaccharide bioactivity can be understood as a modification–structure–function continuum. Physical and processing-assisted approaches, chemical derivatization, and biological fermentation remodel key structural features, including molecular weight, monosaccharide composition, glycosidic linkages, branching, conformation, charge density, and functional groups. These structural changes subsequently influence solubility, bioavailability, receptor interaction, microbial utilization, and host responses, thereby contributing to improved immunomodulatory, anti-inflammatory, metabolic regulatory, gut barrier-protective, microbiota-modulating, and neuroprotective effects.

Several contributions emphasize that extraction and processing conditions can determine both polysaccharide structure and biological outcome. Li et al. used acid-assisted extraction to obtain an *Asparagus cochinchinensis* polysaccharide with defined molecular weight, monosaccharide composition, and glycosidic linkages. In SAMP8 mice, this polysaccharide improved cognitive-related outcomes, reduced oxidative stress and brain pathology, and modulated gut microbiota composition, suggesting a potential role in the microbiota–gut–brain axis. This work illustrates how extraction strategy can produce structurally distinctive polysaccharides with disease-relevant bioactivity.

Biological modification was represented by Yang et al., who showed that *Lactobacillus plantarum* M616 fermentation altered the chemical composition, molecular weight distribution, and monosaccharide ratio of Chinese yam polysaccharides. Importantly, the fermented polysaccharide displayed stronger anti-inflammatory activity than the unfermented counterpart in LPS-induced RAW264.7 macrophages, as reflected by regulation of oxidative stress markers and inflammatory mediators. The study supports microbial fermentation as a green and effective method to modify plant polysaccharides while simultaneously improving biological activity.

The interaction between polysaccharide-derived molecules, gut microbiota, metabolism, and aging was examined by Liu et al. using chitosan oligosaccharide supplementation in a natural aging model. Their study linked COS intervention to remodeling of gut microbial composition, enrichment of *Muribaculaceae*, propionate- and amino acid-associated metabolic changes, cytokine modulation, and reduced p53/p21-related senescence signals. These findings reinforce the importance of integrating microbiome, metabolome, and immune readouts when evaluating modified or low-molecular-weight polysaccharide derivatives.

Chemical modification is a central theme of this Research Topic. Zhao et al. prepared selenium-enriched polysaccharides from blackened jujube pomace using an optimized HNO_3_-Na_2_SeO_3_ route. Selenylation reduced molecular weight, increased crystallinity, and generated distinct Se(IV) coordination patterns, while enhancing immunomodulatory effects in cyclophosphamide-induced immunosuppressed mice. This work is particularly valuable because it combines structural characterization, computational interpretation, biological validation, and byproduct valorization, thereby linking polysaccharide engineering with sustainable functional food development.

Similarly, Wang et al. systematically prepared carboxymethylated, acetylated, sulfated, and phosphorylated derivatives of *Ceratocarpus arenarius* polysaccharides. The modified derivatives showed altered physicochemical properties and enhanced anti-inflammatory activity compared with the native polysaccharide, with sulfation producing the most pronounced inhibition of inflammatory mediators. This study provides a direct comparison of modification strategies and demonstrates that the type of introduced functional group strongly affects bioactivity.

Gao et al. isolated *Phragmites communis* Trin polysaccharide fractions using cellulase-assisted ultrasonication and showed that the uronic acid-enriched, hyperbranched PTP-2 fraction exhibited stronger anti-diabetic potential, including α-glucosidase inhibition, enhanced glucose uptake, glycogen synthesis, and ROS reduction in insulin-resistant HepG2 cells. This work highlights how fractionation and structural differentiation can identify polysaccharide candidates with multi-target metabolic activity.

Collectively, these articles show that polysaccharide modification should be understood as a structure-guided and function-oriented strategy. Molecular weight regulation, branching remodeling, charge introduction, selenium incorporation, microbial fermentation, and controlled extraction can each influence solubility, conformation, receptor interaction, gut microbial utilization, epithelial function, and immune-metabolic responses. Future research should move toward standardized characterization, accurate determination of degree of substitution, batch-to-batch reproducibility, safety and bioavailability assessment, and *in vivo* or clinical validation. Mechanistic studies combining glycomics, multi-omics, molecular docking, receptor assays, microbiota transfer, and advanced structure–function modeling will further strengthen the translational value of modified polysaccharides.

In conclusion, this Research Topic demonstrates that modification is not merely a technical means to improve polysaccharide yield or solubility, but a powerful approach for designing next-generation bioactive polysaccharides. By connecting structural remodeling with biological mechanisms and practical applications, these studies provide a valuable foundation for the development of polysaccharide-based functional foods, nutraceuticals, and therapeutic candidates.

